# Association of lifestyle factors with blood lipids and inflammation in adults aged 40 years and above: a population-based cross-sectional study in Taiwan

**DOI:** 10.1186/s12889-019-7686-0

**Published:** 2019-10-22

**Authors:** Miriam Adoyo Muga, Patrick Opiyo Owili, Chien-Yeh Hsu, Jane C.-J. Chao

**Affiliations:** 1 Department of Human Nutrition and Dietetics, School of Medicine and Health Sciences, Kabarak University, Kabarak, Nakuru, Kenya; 2 Department of Public Health, School of Health Sciences, University of Eastern Africa, Baraton, Eldoret, Kenya; 3 Master Programs in Public Health and Global Health, School of Health Sciences, University of Eastern Africa, Baraton, Eldoret, Kenya; 4 Department of Information Management, National Taipei University of Nursing and Health Sciences, Taipei, Taiwan; 5 Master Program in Global Health and Development, College of Public Health, Taipei Medical University, Taipei, Taiwan; 6 School of Nutrition and Health Sciences, College of Nutrition, Taipei Medical University, 250 Wu-Hsing Street, Taipei, 110 Taiwan; 7 Nutrition Research Center, Taipei Medical University Hospital, Taipei, Taiwan

**Keywords:** Lifestyle factors, Blood lipids, Inflammation, Cardiovascular disease

## Abstract

**Background:**

Lifestyle factors were associated with an increased risk of cardiovascular disease (CVD) occurrence. We explored the associations between lifestyle factors and CVD risk factors, and assessed the interactive effects of lifestyle factors on CVD risk factors.

**Methods:**

A cross-sectional data of 114,082 (57,680 men and 56,402 women) middle-aged adults and elderly in Taiwan were collected from 2001 to 2010. Logistic regression analysis was used to assess the associations between lifestyle factors and CVD risk factors. The relative excess risk due to interaction (RERI) and the attributable proportion due to interaction were used to explore the interactive effect of lifestyle factors on CVD risk factors.

**Results:**

The interaction between alcohol consumption and smoking exhibited an excess risk of high triglycerides (RERI = 0.21; 95% CI: 0.14–0.29), and that of alcohol consumption and physical activity had an excess risk of high LDL-cholesterol (RERI = 0.11; 95% CI: 0.06–0.16) and high blood glucose (RERI = 0.05; 95% CI: 0.01–0.11). Alcohol consumption and vegetable-rich diet (intake of high vegetables with no or low meat) had an excess risk of high LDL-cholesterol and low HDL-cholesterol, but a reduced risk of high triglycerides (RERI = − 0.10; 95% CI: − 0.17 – -0.04). Smoking and physical activity had an increased risk of high blood glucose and a reduced risk of low HDL-cholesterol. Smoking and vegetable-rich diet reduced the risk of high triglycerides (RERI = − 0.11; 95% CI: − 0.18 – − 0.04), high blood glucose (RERI = − 0.14; 95% CI: − 0.21 – − 0.07) and low HDL-cholesterol (RERI = − 0.10; 95% CI: − 0.19 – -0.01).

**Conclusions:**

The interaction between smoking, alcohol consumption, physical activity and diet were associated with lipid profile and blood glucose, hence there was an interaction between these lifestyle factors in an additive scale. Public health promotion should therefore consider multifaceted promotional activities that are likely to make a positive impact on the health status of the Taiwanese population.

## Background

In recent years, heart disease has become the second leading cause of death after malignant neoplasms in Taiwan [1]. Moreover, the prevalence of overweight and obesity among adults in Taiwan increased obviously from 33.2% during 1993–1996 to 43.0% during 2013–2014 [2]. Unhealthy lifestyle habits such as smoking, alcohol consumption, physical inactivity and poor dietary habits have been considered to be associated with an increased risk of overweight and cardiovascular disease (CVD) [3, 4].

Low level of high-density lipoprotein-cholesterol (HDL-C) and raised levels of triglycerides (TG), total cholesterol (TC), low-density lipoprotein-cholesterol (LDL-C), C-reactive protein (CRP) and high blood glucose have been found to be the risk factors of CVD [5–8]. In addition, hypertension and obesity have been consistently associated with the risk factors of CVD [7, 9]. However, good lifestyle habits such as regular exercise and healthy dietary habits have been known as protective factors against hyperlipidemia as well as CVD occurrence and mortality [10–14], while cigarette smoking is known to have a negative effect on blood lipids [15–17]. The association between lifestyle factors and etiology of lipid abnormalities has not been fully understood, partly because of the limitations of study design and/or analytical strategies used. Moreover, the interactive effects of these lifestyle factors on blood lipids and inflammation are not clear in literature.

Therefore, our study assessed the associations between lifestyle factors which include smoking, alcohol consumption, physical activity and dietary habit and CVD biochemical risk factors which include TG, TC, LDL-C, HDL-C, CRP and blood glucose among middle-aged adults and elderly in Taiwan. Further, we explored the interactive effects of lifestyle factors on CVD risk factors in the middle-aged and elderly population.

## Methods

### Study design and population

We used a cross-sectional data collected by Mei Jau (MJ) Health Management Institute and screening centers in Taiwan between 2001 and 2010. The MJ group is a private health screening organization offering comprehensive health screening services and healthcare management consultations. The participants used in this study were individuals who visited the facilities between 2001 and 2010. The participants had answered a structured questionnaire prior to physical examination and signed an informed consent to use their data for research purposes without disclosure of personal information. A total of 765,064 adults aged ≥40 years visited MJ health screening centers in Taipei, Taoyuan, Taichung or Kaohsiung in a 10-year period. If the participants visited MJ health screening centers more than once with multiple entries (*n* = 404,829), we only used the data at their first visit because they had repeated measurements of biomarkers which were excluded. We also excluded those who had some diseases (cancer, diabetes, renal or liver diseases) (*n* = 77,160), certain drug use (psychiatric or hypolipidemic drugs) (*n* = 43,713) or missing data (*n* = 125,280). Therefore, a total of 114,082 (57,680 men, 50.6%; 56,402 women, 49.4%) middle-aged adults and elderly aged ≥40 years participated in the study. The participants with these diseases and those who were taking lipid lowering drugs were excluded because the medication would confound the findings in this study. The exclusion criteria minimized the biases associated with adjustment of lifestyle as a result of being aware of their health status. The demographic and lifestyle data of the participants were collected using standardized and validated questionnaires, and biochemical data were analyzed from collected blood specimens of the participants.

### Ethical consideration

An informed consent was signed by the participants before health screening to agree that anonymized data would be used only for academic purpose. This study was approved by the Taipei Medical University-Joint Institutional Review Board.

### Outcomes: biochemical measurements

Before drawing blood from the participants, overnight fasting (12–14 h) was obligatory. Blood specimens were analyzed at the central laboratory of MJ. Reagents from Randox Laboratories Limited were used to measure the levels of fasting blood glucose and blood lipids (TG, TC and HDL-C). Blood LDL-C levels were determined by using the Friedewald formula (LDL-C = TC – HDL-C – TG/5) [18]. The reagent from Fortress Diagnostics was used to measure CRP levels. Repeated blood tests were performed to ensure accuracy. We used clinically defined levels to identify the participants at a potential high risk of CVD as follows: TG ≥ 150 mg/dL (1.695 mmol/L), TC ≥ 200 mg/dL (5.18 mmol/L), LDL-C ≥ 100 mg/dL (2.59 mmol/L), HDL-C ≤ 40 mg/dL (1.036 mmol/L), CRP ≥ 1.0 mg/L (9.52 nmol/L) or fasting blood glucose ≥100 mg/dL (5.556 mmol/L) [19, 20].

### Predictors: lifestyle factors

Information of lifestyle factors including smoking, alcohol consumption, physical activity and dietary habits were collected using the questionnaire when the participants visited the screening center before health screening. Smoking was categorized as non-smoker, often inhale secondhand smoke, quit smoking in less than 1 year and smoker. Alcohol consumption was divided as not drinking, ≤ 1 glass/week, 2–3 glasses/week and ≥ 4 glasses/week. Physical activity including any form of physical exercise was categorized as none, ≤ 2 h/week, 3–6 h/week and ≥ 7 h/week. Dietary habits were categorized into 3 groups: (1) intake of no or low vegetables with high meat, (2) intake of moderate vegetables with moderate meat and (3) intake of high vegetables with no or low meat. The dietary servings and frequency (i.e., per week or per day) was assessed using the food frequency questionnaire (FFQ) with 22 food items [21]. For example, intake of vegetables and root crops was assessed using the number of bowls per day (i.e., a bowl equals 11 cm in diameter). On the other hand, intake of fruits was assessed using servings per day. The other food items including meat and organ meats were assessed using servings per week. Vegetables or meat dietary pattern was determined by principal component analysis from the 22 food groups without overlapping. The food scores of each dietary pattern were summed up from 1 to 5 defined as the lowest to highest intake frequency of each food group, and intake of each dietary pattern was then divided into tertiles of different consumption indicating no/low intake, moderate intake or high intake.

### Potential confounders: other factors

Other factors included in our analyses were gender, age groups (40–44, 45–49, 50–54, 55–59, 60–64 and ≥ 65 years), education (primary school, high school and college) and marital status (single, married and widowed or divorced). The health characteristics included history of CVD (no or yes), systolic blood pressure (normal or elevated systolic blood pressure ≥ 120 mmHg) and diastolic blood pressure (normal or elevated diastolic blood pressure ≥ 80 mmHg) [22]. Weight status was defined as body mass index (BMI) < 24 kg/m^2^ for non-overweight/obese or BMI ≥ 24 kg/m^2^ for overweight/obese using national criteria developed by the Ministry of Health and Welfare in Taiwan [23], and waist circumference was measured in centimeter. All these sociodemographic factors and health characteristics were considered potential confounders.

### Statistical analysis

The statistical analyses included descriptive, analytical and interactive analyses. The characteristics of lifestyle factors were compared for different CVD risk factors by using chi-square test for categorical data and linear regression analysis for continuous data. Logistic regression analysis was used to assess the association between lifestyle factors and CVD risk factors. Two models were analyzed to produce the odds ratios (ORs) with 95% confidence interval (CI); model 1 was unadjusted and model 2 was adjusted for all the confounders in our study (i.e., sociodemographic and health factors).

The interactive effect of lifestyle factors on CVD risk factors in the additive scale was determined by relative excess risk due to interaction (RERI) and attributable proportion due to interaction (AP) to estimate the risk ratio from the adjusted ORs. Information on RERI and AP has been published elsewhere [24–26]. Each lifestyle factor was dichotomized: smoking vs not smoking, drinking alcohol vs not drinking, physical activity vs no physical activity and high vegetable consumption vs no or low vegetable consumption. The RERI estimates the extra risk due to interaction (RERI ≈ OR_11_− OR_10_ − OR_01_ + 1) [27], when - RERI < 0 suggests less than additivity or a negative interaction between the dependent and independent variables. For instant, if one independent variable combines alcohol consumption or smoking status, there could be an extra risk or a negative effect on the dependent variable. The proportion of a combined effect due to interaction was estimated by using AP (AP = RERI/OR_11_), when – AP <  0 also suggests less than additivity. Logistic regression matrix used with the ‘*ici*’ command in the Stata was to estimate the interaction effect and the 95% CI. The variance and covariance of the coefficients were used to estimate the 95% CI because of our large sample size [28, 29]. All the analyses were performed by the Stata version 13.1 (StataCorp LLC, College Station, Texas) [30]. The *p* ≤ 0.05 level was set for statistical significance.

## Results

### Participants’ characteristics

Table 1 presents the characteristics of the participants by lifestyle factors. All the characteristics were significantly different (*p* <  0.05) between dichotomized lifestyle factors, except for the history of CVD between dichotomized smoking status (*p* = 0.262) and drinking alcohol status (*p* = 0.640), CRP levels between dichotomized physical activity (*p* = 0.430) and diastolic blood pressure between dichotomized vegetable diet (*p* = 0.288).

**Table 1:** Characteristics of participants by lifestyle factors

	Lifestyle factors, *n* = 114,082							
	Smoking		Drinking alcohol		Physical activity		High vegetable diet	
	*n* (%)	*P*-value	*n* (%)	*P*-value	*n* (%)	*P*-value	*n* (%)	*P*-value
All	33,108 (29.0)		30,264 (26.5)		52,305 (45.9)		62,089 (54.4)	
Triglycerides		**< 0.001**		**< 0.001**		**< 0.001**		**< 0.001**
Normal	22,446 (25.9)		21,174 (24.4)		39,375 (45.4)		48,361 (55.7)	
High (≥ 1.695 mmol/L)	10,662 (39.0)		9090 (33.3)		12,930 (47.4)		13,728 (50.3)	
Total cholesterol		**< 0.001**		**0.013**		**< 0.001**		**< 0.001**
Normal	16,921 (28.6)		15,514 (26.2)		27,569 (46.6)		32,838 (55.5)	
High (≥ 5.18 mmol/L)	16,187 (29.5)		14,750 (26.9)		24,736 (45.1)		29,251 (53.3)	
LDL-C		**< 0.001**		**0.003**		**0.016**		**< 0.001**
Normal	8178 (26.6)		7965 (25.9)		13,923 (45.3)		17,571 (57.1)	
High (≥ 2.59 mmol/L)	24,930 (29.9)		22,299 (26.8)		38,382 (46.1)		44,518 (53.4)	
HDL-C		**< 0.001**		**< 0.001**		**< 0.001**		**< 0.001**
Normal	26,767 (26.7)		25,406 (25.3)		44,382 (44.2)		55,507 (55.3)	
High (≥ 1.036 mmol/L)	6341 (46.3)		4858 (35.5)		7923 (57.9)		6582 (48.1)	
CRP		**< 0.001**		**< 0.001**		0.430		**0.014**
Normal	32,012 (28.9)		29,321 (26.4)		50,851 (45.8)		60,456 (54.5)	
High (≥ 9.52 nmol/L)	1096 (35.1)		943 (30.2)		1454 (46.5)		1633 (52.3)	
Fasting blood glucose		**< 0.001**		**< 0.001**		**< 0.001**		**< 0.001**
Normal	15,478 (22.8)		15,842 (23.3)		29,631 (43.6)		38,955 (57.3)	
High (≥ 5.556 mmol/L)	17,630 (38.3)		14,422 (31.3)		22,674 (49.3)		23,134 (50.3)	
Gender		**< 0.001**		**< 0.001**		**< 0.001**		**< 0.001**
Female	5449 (9.7)		6419 (11.4)		24,832 (44.0)		32,767 (58.1)	
Male	27,659 (48.0)		23,845 (41.3)		27,471 (47.9)		29,322(50.8)	
Age group (years)		**< 0.001**		**< 0.001**		**< 0.001**		**< 0.001**
40–44	10,111 (31.4)		8060 (25.0)		13,934 (43.3)		14,875 (46.2)	
45–49	7296 (29.9)		6652 (27.3)		10,930 (44.8)		12,881 (52.8)	
50–54	5151 (28.2)		5065 (27.7)		8363 (45.8)		10,331 (56.6)	
55–59	3709 (26.3)		3788 (26.8)		6168 (43.7)		8458 (59.9)	
60–64	2934 (26.4)		3017 (27.1)		5741 (51.6)		6708 (60.3)	
≥ 65	3907 (28.0)		3682 (26.4)		7169 (51.3)		8836 (63.3)	
Education		**< 0.001**		**< 0.001**		**< 0.001**		**< 0.001**
Primary school	6722 (26.3)		6450 (25.3)		13,284 (52.1)		13,875 (54.4)	
High school	11,785 (33.0)		11,167 (31.3)		16,734 (46.9)		18,458 (51.7)	
College	14,601 (27.6)		12,647 (23.9)		22,287 (42.3)		29,756 (56.3)	
Marital status		**< 0.001**		**< 0.001**		**< 0.001**		**< 0.001**
Single	950 (21.9)		761 (17.5)		1417 (32.7)		2283 (52.6)	
Married	29,337 (30.1)		26,777 (27.5)		45,187 (46.4)		52,807 (54.3)	
Widow/Divorced	2821 (22.7)		2726 (22.0)		5701 (46.0)		6999 (56.4)	
History of CVD		0.262		0.640		**< 0.001**		**< 0.001**
No	31,814 (29.1)		29,064 (26.5)		49,956 (45.6)		59,459 (54.3)	
Yes	1294 (28.3)		1200 (26.2)		2349 (51.3)		2630 (54.4)	
Weight status		**< 0.001**		**< 0.001**		**0.003**		**< 0.001**
Non-overweight/obese	20,656 (26.4)		18,947 (24.3)		35,591 (45.6)		44,286 (56.7)	
Overweight/obese	12,452 (34.6)		11,317 (31.5)		16,714 (46.5)		17,803 (49.5)	
Waist circumference (cm), M (SD)^a^	83.0 (9.2)	**< 0.001**	82.7 (9.2)	**< 0.001**	79.4 (9.5)	**< 0.001**	78.3 (9.4)	**< 0.001**
Systolic blood pressure		**0.005**		**< 0.001**		**< 0.001**		**< 0.001**
Normal	16,081 (28.6)		13,628 (24.3)		24,359 (43.4)		30,176 (53.7)	
≥ 120 mmHg	17,027 (29.4)		16,636 (28.7)		27,946 (48.2)		31,913 (55.1)	
Diastolic blood pressure		**< 0.001**		**< 0.001**		**< 0.001**		0.288
Normal	22,378 (27.8)		19,495 (24.3)		36,204 (45.0)		43,843 (54.5)	
≥ 80 mmHg	10,730 (31.9)		10,769 (32.0)		16,101 (47.8)		18,246 (54.2)	

^a^ Linear regression analysis was used to determine the mean (M) difference and standard deviation (SD)

LDL-C, low-density lipoprotein-cholesterol; HDL-C, high-density lipoprotein-cholesterol; CRP, C-reactive protein; CVD, cardiovascular disease

The *p*-values ≤0.05 are presented in bold

### Associations between lifestyle factors and CVD risk factors

The unadjusted and adjusted ORs of CVD biomarkers by lifestyle factors are shown in Table 2. Significant associations between lifestyle factors and most of CVD risk factors were observed in both unadjusted and adjusted models. The adjusted model revealed that smoking significantly increased the development of high TG (OR = 1.44; 95% CI: 1.38–1.50; *p* ≤ 0.001), TC (OR = 1.08; 95% CI: 1.04–1.12; *p* ≤ 0.001), CRP (OR = 1.33; 95% CI: 1.19–1.47; *p* ≤ 0.001), blood glucose (OR = 1.49; 95% CI: 1.43–1.55; *p* ≤ 0.001) and low HDL-C levels (OR = 1.60; 95% CI: 1.52–1.68; *p* ≤ 0.001) compared with non-smoking. Additionally, those who quitted smoking attenuated all the CVD risk factors, except for blood TG level (OR = 1.06; 95% CI: 1.01–1.12; *p* ≤ 0.05). Drinking alcohol (≥ 4 glasses/week) was more likely to have high TG (OR = 1.25; 95% CI: 1.18–1.33; *p* ≤ 0.001), TC (OR = 1.14; 95% CI: 1.08–1.20; *p* ≤ 0.001) and CRP levels (OR = 1.22; 95% CI: 1.05–1.42; *p* ≤ 0.01), but less likely to have high LDL-C (OR = 0.79; 95% CI: 0.74–0.85; *p* ≤ 0.001), blood glucose (OR = 0.68; 95% CI: 0.64–0.72; *p* ≤ 0.001) and low HDL-C levels (OR = 0.64; 95% CI: 0.59–0.69; *p* ≤ 0.001) than non-drinking. Those who were engaged in physical activity for more than 7 h per week were less likely to have high TG (OR = 0.86; 95% CI: 0.81–0.91; *p* ≤ 0.001), TC (OR = 0.88; 95% CI: 0.84–0.93; *p* ≤ 0.001) and CRP levels (OR = 0.79; 95% CI: 0.68–0.92; *p* ≤ 0.01), but were more likely to have low HDL-C level (OR = 1.45; 95% CI: 1.35–1.57; *p* ≤ 0.001) and high blood glucose level (OR = 1.07; 95% CI: 1.01–1.13; *p* ≤ 0.05) than those who were not. Those who had a diet high in vegetables and no/low meat were less likely to have high TG (OR = 0.94; 95% CI: 0.91–0.97; *p* ≤ 0.001), TC (OR = 0.85; 95% CI: 0.83–0.88; *p* ≤ 0.001), LDL-C (OR = 0.81; 95% CI: 0.78–0.84; *p* ≤ 0.001) and blood glucose levels (OR = 0.84; 95% CI: 0.81–0.87; *p* ≤ 0.001), and less likely to have low HDL-C level (OR = 0.92; 95% CI: 0.88–0.97; *p* ≤ 0.001) than those who had high meat consumption and no/low vegetables.

**Table 2:** Unadjusted and adjusted odd ratios and 95% confidence interval of cardiovascular disease risk factors by lifestyle factors

	Odds ratio (95% confidence interval)					
	TG ≥ 1.695 mmol/L	TC ≥ 5.18 mmol/L	LDL-C ≥ 2.59 mmol/L	HDL-C ≤ 1.036 mmol/L	CRP ≥ 9.52 nmol/L	BG ≥ 5.556 mmol/L
Unadjusted model						
Smoking (Ref: Non-smoker)						
Inhale secondhand smoke	1.08 (1.01, 1.16)*	1.06 (1.00, 1.13)*	1.05 (0.98, 1.12)	1.22 (1.11, 1.35)***	1.00 (0.83, 1.21)	1.11 (1.04, 1.18) ***
Quit smoking	1.70 (1.62, 1.78)***	1.06 (1.01, 1.11)*	1.26 (1.20, 1.33)***	1.92 (1.80, 2.04)***	1.31 (1.15, 1.49)***	2.02 (1.93, 2.11) ***
Smoker	2.10 (2.03, 2.17)***	1.03 (1.01, 1.07)*	1.18 (1.14, 1.22)***	2.88 (2.76, 3.00)***	1.42 (1.30, 1.55)***	2.48 (2.41, 2.56) ***
Drinking alcohol (Ref: Not drinking)						
≤ 1 glass/week	1.22 (1.17, 1.26)***	0.98 (0.95, 1.01)	1.06 (1.02, 1.10)**	1.58 (1.51, 1.66)***	1.09 (0.98, 1.21)	1.35 (1.30, 140) ***
2–3 glasses/week	1.77 (1.68, 1.86)***	1.08 (1.03, 1.13)**	1.02 (0.97, 1.08)	1.60 (1.50, 1.71)***	1.26 (1.10, 1.44)***	1.63 (1.55, 1.70)***
≥ 4 glasses/week	2.39 (2.26, 2.52)***	1.15 (1.09, 1.21)***	1.04 (0.98, 1.10)	1.77 (1.65, 1.90)***	1.46 (1.27, 1.68)***	1.84 (1.75, 1.94)***
Physical activity (Ref: None)						
≤ 2 h/week	1.14 (1.10, 1.18)***	0.91 (0.89, 0.94)***	1.02 (0.98, 1.05)	1.86 (1.78, 1.94)***	1.08 (0.99, 1.18)	1.29 (1.25, 1.32)***
3–6 h/week	1.06 (1.02, 1.10)**	0.96 (0.93, 0.98)**	1.03 (0.99, 1.07)	1.67 (1.59, 1.75)***	0.97 (0.87, 1.07)	1.25 (1.21, 1.29)***
≥ 7 h/week	0.96 (0.90, 1.01)	0.99 (0.95, 1.04)	1.10 (1.04, 1.16)***	1.47 (1.37, 1.58)***	0.99 (0.85, 1.15)	1.19 (1.13, 1.25)***
Diet (Ref: No/low vegetables + high meat)						
Moderate (vegetables + meat)	0.87 (0.84, 0.90)***	0.95 (0.93, 0.98)**	0.91 (0.88, 0.94)***	0.81 (0.78, 0.85)***	0.89 (0.81, 0.97)**	0.83 (0.81, 0.86)***
High vegetables + no/low meat	0.73 (0.71, 0.76)***	0.88 (0.85, 0.90)***	0.80 (0.78, 0.83)***	0.67 (0.64, 0.70)***	0.88 (0.81, 0.96)**	0.67 (0.65, 0.70)***
Adjusted model^a^						
Smoking (Ref: Non-smoker)						
Inhale secondhand smoke	0.97 (0.90, 1.05)	1.05 (0.99, 1.12)	1.04 (0.97, 1.12)	1.12 (1.01, 1.24)*	0.97 (0.80, 1.18)	1.05 (0.98, 1.12)
Quit smoking	1.06 (1.01, 1.12)*	1.04 (0.99, 1.10)	1.02 (0.96, 1.08)	0.98 (0.91, 1.04)	1.11 (0.97, 1.28)	1.03 (0.98, 1.08)
Smoker	1.44 (1.38, 1.50)***	1.08 (1.04, 1.12)***	1.03 (0.99, 1.08)	1.60 (1.52, 1.68)***	1.33 (1.19, 1.47)***	1.49 (1.43, 1.55)***
Drinking alcohol (Ref: Not drinking)						
≤ 1 glass/week	0.88 (0.85, 0.92)***	0.98 (0.94, 1.01)	0.90 (0.87, 0.94)***	0.83 (0.79, 0.88)***	0.93 (0.84, 1.04)	0.78 (0.75, 0.81)***
2–3 glasses/week	1.01 (0.96, 1.07)	1.06 (1.01, 1.12)*	0.79 (0.75, 0.84)***	0.63 (0.59, 0.68)***	1.04 (0.90, 1.20)	0.67 (0.63, 0.71)***
≥ 4 glasses/week	1.25 (1.18, 1.33)***	1.14 (1.08, 1.20)***	0.79 (0.74, 0.85)***	0.64 (0.59, 0.69)***	1.22 (1.05, 1.42)**	0.68 (0.64, 0.72)***
Physical activity (Ref: None)						
≤ 2 h/week	1.11 (1.07, 1.15)***	0.92 (0.89, 0.95)***	1.03 (0.99, 1.06)	1.94 (1.85, 2.03)***	1.10 (1.01, 1.21)*	1.34 (1.29, 1.38)***
3–6 h/week	1.04 (1.00, 1.08)*	0.93 (0.90, 0.96)***	1.00 (0.96, 1.04)	1.73 (1.65, 1.82)***	0.93 (0.84, 1.03)	1.25 (1.20, 1.29)***
≥ 7 h/week	0.86 (0.81, 0.91)***	0.88 (0.84, 0.93)***	0.97 (0.91, 1.02)	1.45 (1.35, 1.57)***	0.79 (0.68, 0.92)**	1.07 (1.01, 1.13)*
Diet (Ref: No/low vegetables + high meat)						
Moderate (vegetables + meat)	0.97 (0.94, 1.01)	0.95 (0.92, 0.97)***	0.91 (0.88, 0.94)***	0.93 (0.89, 0.97)**	0.91 (0.83, 0.99)*	0.91 (0.88, 0.94)***
High vegetables + no/low meat	0.94 (0.91, 0.97)***	0.85 (0.83, 0.88)***	0.81 (0.78, 0.84)***	0.92 (0.88, 0.97)***	0.92 (0.84, 1.01)	0.84 (0.81, 0.87)***

^a^The model was adjusted for smoking, drinking, physical activity, diet, gender, age, education, marital status, history of cardiovascular disease, waist circumference, systolic blood pressure and diastolic blood pressure

*TG* Triglycerides, *TC* Total cholesterol, *LDL-C* Low-density lipoprotein-cholesterol, *HDL-C* High-density lipoprotein-cholesterol, *CRP* C-reactive protein, *BG* Blood glucose

*p ≤ 0.05; **p ≤ 0.01; ***p ≤ 0.001

### Interactive effects of lifestyle factors on CVD risk factors

Table 3 indicates the adjusted ORs of CVD biomarkers by interaction of lifestyle factors. Participants who drank and smoked were more likely to have high TG (OR = 1.29; 95% CI: 1.24–1.35; *p* ≤ 0.001), TC (OR = 1.08; 95% CI: 1.04–1.12; *p* ≤ 0.001) and CRP levels (OR = 1.24; 95% CI: 1.11–1.38; *p* ≤ 0.001), and less likely to have high LDL-C (OR = 0.83; 95% CI: 0.79–0.86; *p* ≤ 0.001) and blood glucose levels (OR = 0.91; 95% CI: 0.88–0.95; *p* ≤ 0.001) than those who neither drank nor smoked. Smoking and drinking negatively interacted with TC, LDL-C, HDL-C and blood glucose levels (*p* ≤ 0.001), but had an excess risk of high TG level (RERI = 0.21; 95% CI: 0.14–0.29; *p* ≤ 0.001) due to the interaction.

**Table 3:** Adjusted ORs of CVD biomarkers by lifestyle factors, relative excess risk due to interaction (RERI) and attributable proportion due to interaction (AP)

Interaction	Total *n*	TG ≥ 1.695 mmol/L		TC ≥ 5.18 mmol/L		LDL-C ≥ 2.59 mmol/L		HDL-C ≤ 1.036 mmol/L		CRP ≥ 9.52 nmol/L		BG ≥ 5.556 mmol/L	
		*n*	OR (95% CI) ^a^	*n*.	OR (95% CI) ^a^	*n*	OR (95% CI) ^a^	*n*	OR (95% CI) ^a^	*n*	OR (95% CI) ^a^	*n*	OR (95% CI) ^a^
Drinking by smoking													
Not drinking													
Not smoking	67,939	13,574	1.00	32,310	1.00	48,687	1.00	5752	1.00	1667	1.00	22,999	1.00
Smoker	15,879	4672	1.18 (1.13, 1.23)***	7840	1.12 (1.08, 1.16)***	12,336	1.16 (1.11, 1.21)***	3085	1.38 (1.31, 1.46)***	514	1.23 (1.10, 1.37)***	8615	1.34 (1.29, 1.40)***
Drinking													
Not smoking	13,035	3100	0.90 (0.86, 0.95)***	6403	1.07 (1.03, 1.11)***	9705	0.98 (0.93, 1.02)	1602	0.80 (0.75, 0.85)***	361	1.03 (0.92, 1.67)	5407	0.80 (0.77, 0.84)***
Smoker	17,229	5990	1.29 (1.24, 1.35)***	8347	1.08 (1.04, 1.12)***	12,594	0.83 (0.79, 0.86)***	3256	0.98 (0.93, 1.04)	582	1.24 (1.11, 1.38)***	9015	0.91 (0.88, 0.95)***
*RERI*_OR_ (95% CI)			0.21 (0.14, 0.29)***		−0.11 (−0.17, − 0.05)***		−0.31 (− 0.38, − 0.24)***		− 0.20 (− 0.29, − 0.11)***		− 0.03 (− 0.22, 0.17)		− 0.23 (− 0.30, − 0.17)***
*AP*_OR_ (95% CI)			0.16 (0.11, 0.22)***		− 0.10 (− 0.16, − 0.04)***		−0.37 (− 0.46, − 0.28)***		−0.20 (− 0.30, − 0.11)***		−0.02 (− 0.18, 0.13)		− 0.25 (− 0.33, − 0.18)***
Drinking by physical activity													
Not drinking													
No physical activity	49,275	10,522	1.00	23,923	1.00	35,864	1.00	4254	1.00	1269	1.00	17,815	1.00
Physical activity	34,543	7697	1.06 (1.02, 1.09)**	16,227	0.91 (0.89, 0.94)***	25,159	0.98 (0.95, 1.01)	4583	1.77 (1.69, 1.85)***	912	0.98 (0.90, 1.07)	13,799	1.22 (1.18, 1.26)***
Drinking													
No physical activity	12,502	3857	1.01 (0.96, 1.06)	6241	1.02 (0.97, 1.06)	9076	0.80 (0.79, 0.84)***	1518	0.71 (0.66, 0.75)***	401	1.02 (0.90, 1.15)	5547	0.68 (0.65, 0.71)***
Physical activity	17,762	5233	1.01 (0.96, 1.05)	8509	0.94 (0.90, 0.97)***	13,223	0.89 (0.85, 0.93)***	3340	1.33 (1.26, 1.41)***	542	0.98 (0.88, 1.10)	8875	0.95 (0.91, 0.99)*
*RERI*_OR_ (95% CI)			−0.06 (−0.12, 0.01)		0.01 (− 0.04, 0.06)		0.11 (0.06, 0.16)***		−0.14 (− 0.24, − 0.05)**		−0.01 (− 0.17, 0.14)		0.05 (0.01, 0.11) *
*AP*_OR_ (95% CI)			−0.06 (− 0.12, − 0.01)		0.01 (− 0.05, 0.07)		0.12 (0.06, 0.18)***		− 0.11 (− 0.18, − 0.03)**		−0.01 (− 0.17, 0.15)		0.06 (0.01, 0.12)*
Drinking by diet													
Not drinking													
No/low vegetables	35,695	8329	1.00	17,602	1.00	26,676	1.00	4331	1.00	956	1.00	14,737	1.00
High vegetables	48,123	9890	0.98 (0.95, 1.02)	22,548	0.88 (0.86, 0.91)***	34,347	0.85 (0.82, 0.88)***	4506	0.93 (0.88, 0.97)**	1225	0.96 (0.88, 1.05)	16,877	0.88 (0.85, 0.91)***
Drinking													
No/low vegetables	16,298	5252	1.03 (0.98, 1.08)	8047	1.00 (0.96, 1.04)	12,128	0.83 (0.79, 0.87)***	2782	0.72 (0.68, 0.76)***	535	1.05 (0.94, 1.18)	8165	0.73 (0.70, 0.76)***
High vegetables	13,966	3838	0.91 (0.86, 0.95)***	6703	0.92 (0.89, 0.96)***	10,171	0.75 (0.72, 0.79)***	2076	0.70 (0.66, 0.75)***	408	0.93 (0.82, 1.05)	6257	0.65 (0.62, 0.68)***
*RERI*_OR_ (95% CI)			−0.10 (− 0.17, − 0.04)***		0.03 (− 0.01, 0.09)		0.08 (0.03, 0.13)**		0.06 (0.001, 0.13)*		−0.08 (− 0.24, 0.08)		0.04 (− 0.004, 0.09)
*AP*_OR_ (95% CI)			−0.11 (− 0.18, − 0.04)***		0.04 (− 0.01, 0.10)		0.10 (0.04, 0.17)**		0.09 (0.001, 0.18)*		−0.09 (− 0.26, 0.09)		0.06 (− 0.006, 0.13)
Smoking by physical activity													
Not smoking													
No physical activity	44,497	8770	1.00	21,484	1.00	31,981	1.00	3139	1.00	1078	1.00	14,560	1.00
Physical activity	36,477	7877	1.08 (1.04, 1.12)***	17,229	0.93 (0.91, 0.96)***	26,411	1.00 (0.97, 1.03)	4215	1.73 (1.65, 1.83)***	950	1.03 (0.94, 1.12)	13,846	1.24 (1.21, 1.29)***
Smoker													
No physical activity	17,280	5609	1.30 (1.25, 1.36)***	8680	1.10 (1.05, 1.14)***	12,959	1.02 (0.97, 1.06)	2633	1.30 (1.23, 1.38)***	592	1.27 (1.14, 1.42)***	8802	1.25 (1.20, 1.31)***
Physical activity	15,828	5053	1.28 (1.22, 1.34)***	7507	0.96 (0.92, 1.00)	11,971	1.05 (1.00, 1.10)*	3708	2.40 (1.20, 1.51)***	504	1.16 (1.03, 1.31)*	8828	1.61 (1.54, 1.68)***
*RERI*_OR_ (95% CI)			−0.10 (−0.17, −0.03)**		−0.06 (−0.12, −0.02)*		0.04 (− 0.03, 0.10)		0.36 (0.23, 0.49)***		−0.14 (− 0.32, 0.04)		0.11 (0.03, 0.19)**
*AP*_OR_ (95% CI)			−0.08 (− 0.14, − 0.02)**		−0.07 (− 0.13, − 0.02)*		0.03 (− 0.02, 0.09)		0.15 (0.10, 0.20)***		− 0.12 (− 0.28, 0.03)		0.07 (0.02, 0.11)**
Smoking by diet													
Not smoking													
No/low vegetables	33,275	7265	1.00	16,303	1.00	24,614	1.00	3325	1.00	844	1.00	12,538	1.00
High vegetables	47,699	9382	0.98 (0.94, 1.01)	22,410	0.90 (0.87, 0.92)***	33,778	085 (0.82, 0.88)***	4029	0.96 (0.91, 1.00)	1184	0.97 (0.89, 1.06)	15,868	0.90 (0.87, 0.93)***
Smoker													
No/low vegetables	18,718	6316	1.30 (1.24, 1.36)***	9346	1.08 (1.04, 1.12)***	14,190	1.01 (0.96, 1.05)	3788	1.39 (1.31, 1.47)***	647	1.26 (1.12, 1.41)***	10,364	1.33 (1.28, 1.39)***
High vegetables	14,390	4346	1.17 (1.11, 1.23)***	6841	0.95 (0.91, 0.99)*	10,740	0.91 (0.87, 0.95)***	2553	1.24 (1.17, 1.32)***	449	1.11 (0.98, 1.25)	7266	1.09 (1.05, 1.15)***
*RERI*_OR_ (95% CI)			−0.11 (−0.18, −0.04)**		−0.02 (−0.07, 0.02)		0.05 (−0.001, 0.11)		−0.10 (− 0.19, − 0.01)*		−0.12 (− 0.29, 0.05)		− 0.14 (− 0.21, − 0.07)***
*AP*_OR_ (95% CI)			−0.09 (− 0.16, − 0.03)**		−0.03 (− 0.08, 0.03)		0.06 (− 0.001, 0.12)		− 0.08 (− 0.15, − 0.004)*		−0.11 (− 0.27, 0.05)		− 0.13 (− 0.19, − 0.07)***

^a^The model was adjusted for smoking, drinking, physical activity, diet, gender, age, education, marital status, history of cardiovascular disease, waist circumference, systolic blood pressure and diastolic blood pressure

*TG* Triglycerides, *TC* Total cholesterol, *LDL-C* Low-density lipoprotein-cholesterol, *HDL-C* High-density lipoprotein-cholesterol, *CRP* C-reactive protein, *BG* Blood glucose, *RERI* Relative excess risk due to interaction, *AP* Attributable proportion due to interaction

*p ≤ 0.05; **p ≤ 0.01; ***p ≤ 0.001; significant data are presented in bold

Those who drank and were engaged in physical activity were less likely to have higher TC (OR = 0.94; 95% CI: 0.90–0.97; *p* ≤ 0.001), LDL-C (OR = 0.89; 95% CI: 0.85–0.93; *p* ≤ 0.001) and blood glucose levels (OR = 0.95; 95% CI: 0.91–0.99; *p* ≤ 0.05), but more likely to have low HDL-C level (OR = 1.33; 95% CI: 1.26–1.41; *p* ≤ 0.001) than the non-drinkers who were also not engaged in physical activity. Alcohol consumption and physical activity had an excess risk of high LDL-C (RERI = 0.11; 95% CI: 0.06–0.16; *p* ≤ 0.001) and high blood glucose levels (RERI = 0.05; 95% CI: 0.01–0.11; *p* ≤ 0.05) due to the interaction, but had a reduced risk of low HDL-C level (RERI = − 0.14; 95% CI: − 0.24 – 0.05; *p* ≤ 0.01).

Participants who were consuming alcohol and were eating a vegetable-rich diet were less likely to have high TG (OR = 0.91; 95% CI: 0.86–0.95; *p* ≤ 0.001), TC (OR = 0.92; 95% CI: 0.89–0.96; *p* ≤ 0.001), LDL-C (OR = 0.75; 95% CI: 0.72–0.79; *p* ≤ 0.001) and blood glucose levels (OR = 0.65; 95% CI: 0.62–0.68; *p* ≤ 0.001), and less likely to have low HDL-C level (OR = 0.70; 95% CI: 0.66–0.75; *p* ≤ 0.001) than those who drank and had no or low intake of vegetables. Consumption of alcohol and vegetable-rich diet had an excess risk of high LDL-C (RERI = 0.08; 95% CI: 0.03–0.13; *p* ≤ 0.01) and low HDL-C levels (RERI = 0.06; 95% CI: 0.001–0.13; *p* ≤ 0.05) due to the interaction, but a reduced risk of high TG level (RERI = − 0.10; 95% CI: − 0.17 – − 0.04; *p* ≤ 0.001).

Smokers who were engaged in physical activity were more likely to have high TG (OR = 1.28; 95% CI: 1.22–1.34; *p* ≤ 0.001), LDL-C (OR = 1.05; 95% CI: 1.00–1.10; *p* ≤ 0.05), CRP (OR = 1.16; 95% CI: 1.03–1.31; *p* ≤ 0.05) and blood glucose levels (OR = 1.61; 95% CI: 1.54–1.68; *p* ≤ 0.001), and low HDL-C level (OR = 2.40; 95% CI: 1.20–1.51; *p* ≤ 0.001) than non-smokers who did not exercise. The interaction between smoking and physical activity had an excess risk of low HDL-C (RERI = 0.36; 95% CI: 0.23–0.49; *p* ≤ 0.001) and high blood glucose levels (RERI = 0.11; 95% CI: 0.03–0.19; *p* ≤ 0.01), but had a reduced risk of high TG (RERI = − 0.10; 95% CI: − 0.17 – − 0.03; *p* ≤ 0.01) and high TC levels (RERI = − 0.06; 95% CI: − 0.12 – − 0.029; *p* ≤ 0.05). Smokers who consumed a diet high in vegetables were still more likely to have high TG (OR = 1.17; 95% CI: 1.11–1.23; *p* ≤ 0.001), blood glucose (OR = 1.09; 95% CI: 1.05–1.15; *p* ≤ 0.001), and low HDL-C levels (OR = 1.24; 95% CI: 1.17–1.32; *p* ≤ 0.001), but less likely to have high TC (OR = 0.95; 95% CI: 0.91–0.99; *p* ≤ 0.05) and LDL-C levels (OR = 0.91; 95% CI: 0.87–0.95; *p* ≤ 0.001) than non-smokers who consumed a diet with no or low vegetables. Smoking and vegetable-rich diet had a reduced risk of high TG (RERI = − 0.11; 95% CI: − 0.18 – − 0.04; *p* ≤ 0.01), high blood glucose levels (RERI = − 0.14; 95% CI: − 0.21 – − 0.07; *p* ≤ 0.001) and low HDL-C (RERI = − 0.10; 95% CI: − 0.19 – − 0.01; *p* ≤ 0.05) in an additive scale.

## Discussion

Our study explored the associations between lifestyle factors and CVD risk factors, and further assessed the interactive effects of lifestyle factors on CVD risk factors. Consistent with the findings of the previous studies [15–17, 31–33], our study found that smokers were more likely to have high levels of TG, TC, CRP and blood glucose and low HDL-C level, which raises the risk of CVD in smokers. The mechanism of abnormal lipid profiles in smokers may be due to an increased release of catecholamine which causes elevated free fatty acids and leads to a decrease in HDL-C level and a surge in LDL-C level [34]. In the present study, we also found that participants who drank alcohol had elevated levels of TG, TC and CRP, but were less likely to have high LDL-C, blood glucose and low HDL-C levels. The results on TG, LDL-C, blood glucose and HDL-C levels in drinkers are similar to other studies [35, 36]. On the other hand, CRP level in drinkers was dependent on the amount of alcohol consumed as shown in the previous study that CRP level was elevated among heavy drinkers [37]. However, an animal study observed the contrary results regarding the effect of alcohol consumption on blood TG and TC levels [38].

Our results revealed those who had physical activity were more likely to have low TG, TC and CRP levels, which were consistent with the previous studies [12, 39], but were more likely to have low HDL-C level and high blood glucose level. The finding on negative effect of exercise on HDL-C level may be because of exercise intensity and volume, gender disparity and population difference. Exercise intensity and volume required to elevate HDL-C level were more for women than those for men because HDL-C level is usually higher in women than that in men [40]. Higher HDL-C level was found in physically active women compared with that in sedentary women, but not in men [13]. High blood glucose level was found in those who had physical activity, and the result was possibly because of the variations in exercise timing, duration, intensity and type which were not recorded in our study [12, 14]. Additionally, our results supported the findings of other studies that participants with a high vegetable diet were less likely to have high TG, TC, LDL-C and blood glucose levels, and low HDL-C level than those who consumed a high meat diet [41–43].

Our study found that smoking and alcohol consumption had a negative interaction on TC, LDL-C, blood glucose and HDL-C levels. The reduced risk effect by the interaction of smoking and alcohol consumption was predominantly contributed from alcohol per se. Our findings observed those who consumed alcohol without smoking were less likely to have high blood glucose level and low HDL-C level, but smokers who did not consume alcohol were more likely to have high concentrations of all CVD biomarkers and low HDL-C level. However, smoking and alcohol consumption had a positive interaction on TG level, which was possibly attributed to smoking. Our study showed that smokers who did not consume alcohol were more likely to have raised TG level, which was contrary to the result that alcohol consumers who did not smoke were less likely to have high TG level.

Those who consumed alcohol and were engaged in physical activity had an excess risk on LDL-C and blood glucose levels due to the interaction. The reason for the positive interaction between alcohol consumption and physical activity was possibly because of exercise intensity and volume as well as gender disparity discussed earlier [12–14, 40]. However, alcohol consumption and physical activity reduced the risk of low HDL-C level mainly because physically active participants who did not consume alcohol were less likely to have low HDL-C level.

The consumption of alcohol and vegetable-rich diet revealed an extra risk on LDL-C and HDL-C levels, but alcohol or vegetable consumers was less likely to have high LDL-C and low HDL-C levels. The interaction between alcohol and vegetable-rich diet is still not clear. A significant additive effect of alcohol on postprandial TG level was observed as it accompanied a meal high in saturated fat [44]. On the other hand, alcohol had cholesterol-lowering effect as a result of the action of alcohol on the gastrointestinal tract [45]. More studies are still required to explain the interaction between alcohol and vegetable consumption.

We found a positive interaction between smoking and physical activity on HDL-C and blood glucose levels, but a reduced risk on TG and TC levels. Those who smoked but had physical activity were 2.4 times more likely to have low HDL-C level, and 1.6 times more likely to have high blood glucose level. Our results indicated that smokers who had physical activity were 1.3 times more likely to have high TG level. However, the interaction was negative on TG level between smoking and physical activity which needs further studies to explain this finding, though physically active participants who did not smoke had lower TC level. Additionally, smokers who consumed a vegetable-rich diet reduced the risk of high TG and blood glucose levels as well as low HDL-C level possibly due to the consumption of vegetables, which was consistent with our finding that those who consumed a diet high in vegetables and did not smoke were less likely to have high TG and blood glucose levels and low HDL-C level.

### Strengths and limitations

This is the first study, to the best of our knowledge, to explore the interactive effect of lifestyle factors on CVD risk factor in middle-aged adults and elderly. The sample size collected for 10 years was large, and we may be able to generalize the findings to the population of middle-aged adults and elderly in Taiwan. The variables included not only lifestyle factors but several biochemical measurements. However, the cross-sectional data reduced the possibility of causal assertions, and a cohort study or randomized controlled trial design would be appropriate for causal association. Second, our assessment of lifestyle factors may not have been comprehensive enough to adjust for associated confounding factors such as the type and intensity of exercise. Finally, data were collected by one institution that has branches in major cities of Taiwan, but people with different demographic characteristics might not have visited the institution. Therefore, one needs caution in interpreting our results.

## Conclusions

Smoking and drinking are associated with higher blood lipids and CRP. Whereas physical activity is correlated with lower blood lipids and CRP, and vegetable-rich diet is correlated with lower blood lipids and glucose. Smoking, drinking, physical activity and vegetable-rich diet have an additive interaction on blood lipids and glucose. In light of these findings, public health promotion should therefore consider multifaceted promotional activities that are likely to make a positive impact on the health status of the Taiwanese population. Further research is also needed that will include other undetermined lifestyle factors and CVD risk factors, and to understand the mechanisms underlying these interactions of lifestyle factors before developing efficient and effective primary prevention strategies for CVD.

## Data Availability

The data are available from Mei Jau (MJ) Health Management Institute, but restrictedly apply to the availability of these data, and are not publicly available. Data are only available from the authors upon reasonable request and with permission of MJ Health Management Institute.

## Abbreviations

AP: Attributable proportion due to interaction
BG: Blood glucose
BMI: Body mass index
CRP: C-reactive protein
CVD: Cardiovascular disease
HDL-C: High-density lipoprotein-cholesterol
LDL-C: Low-density lipoprotein-cholesterol
RERI: Relative excess risk due to interaction
TC: Total cholesterol
TG: Triglycerides
